# *MAPT* genotype-dependent mitochondrial aberration and ROS production trigger dysfunction and death in cortical neurons of patients with hereditary FTLD

**DOI:** 10.1016/j.redox.2022.102597

**Published:** 2022-12-30

**Authors:** Lisanne Korn, Anna M. Speicher, Christina B. Schroeter, Lukas Gola, Thilo Kaehne, Alexander Engler, Paul Disse, Juncal Fernández-Orth, Júlia Csatári, Michael Naumann, Guiscard Seebohm, Sven G. Meuth, Hans R. Schöler, Heinz Wiendl, Stjepana Kovac, Matthias Pawlowski

**Affiliations:** a Department of Neurology with Institute of Translational Neurology, University Hospital Münster, Münster, Germany; b Institute of Experimental Internal Medicine, Otto-von-Guericke University, Magdeburg, Germany; c Institute for Genetics of Heart Diseases (IfGH), Department of Cardiovascular Medicine, University Hospital Münster, Münster, Germany; d Chemical Biology of Ion Channels (Chembion), GRK 2515, University of Münster, 48149, Münster, Germany; e Department of Pediatric Hematology and Oncology, University Medical Center Freiburg, Germany; f Department of Cell and Developmental Biology, Max-Planck-Institute for Molecular Biomedicine, Münster, Germany; g Medical Faculty, University of Münster, Münster, Germany

**Keywords:** *MAPT* mutation, Cell death, Mitochondria, ROS, FTLD, Forward programming

## Abstract

Tauopathies are a major type of proteinopathies underlying neurodegenerative diseases. Mutations in the tau-encoding *MAPT*-gene lead to hereditary cases of frontotemporal lobar degeneration (FTLD)-tau, which span a wide phenotypic and pathological spectrum. Some of these mutations, such as the N279K mutation, result in a shift of the physiological 3R/4R ratio towards the more aggregation prone 4R isoform. Other mutations such as V337M cause a decrease in the *in vitro* affinity of tau to microtubules and a reduced ability to promote microtubule assembly. Whether both mutations address similar downstream signalling cascades remains unclear but is important for potential rescue strategies. Here, we developed a novel and optimised forward programming protocol for the rapid and highly efficient production of pure cultures of glutamatergic cortical neurons from hiPSCs. We apply this protocol to delineate mechanisms of neurodegeneration in an FTLD-tau hiPSC-model consisting of *MAPT*^N279K^- or *MAPT*^V337M^-mutants and wild-type or isogenic controls. The resulting cortical neurons express *MAPT*-genotype-dependent dominant proteome clusters regulating apoptosis, ROS homeostasis and mitochondrial function. Related pathways are significantly upregulated in *MAPT*^N279K^ neurons but not in *MAPT*^V337M^ neurons or controls. Live cell imaging demonstrates that both *MAPT* mutations affect excitability of membranes as reflected in spontaneous and stimulus evoked calcium signals when compared to controls, albeit more pronounced in *MAPT*^N279K^ neurons. These spontaneous calcium oscillations in *MAPT*^N279K^ neurons triggered mitochondrial hyperpolarisation and fission leading to mitochondrial ROS production, but also ROS production through NOX2 acting together to induce cell death. Importantly, we found that these mechanisms are *MAPT* mutation-specific and were observed in *MAPT*^N279K^ neurons, but not in *MAPT*^V337M^ neurons, supporting a pathological role of the 4R tau isoform in redox disbalance and highlighting *MAPT*-mutation specific clinicopathological-genetic correlations, which may inform rescue strategies in different *MAPT* mutations.

## Highlights

• Human iPSC derived *MAPT*^N279K^ neurons have strongly increased expression of 4R tau.
• Physiological calcium signals, mitochondria and ROS are altered in *MAPT*^N279K^ neurons.
• Mitochondrial and NOX2 derived ROS lead to cell death in *MAPT*^N279K^ neurons.
• Alterations in *MAPT*^N279K^ neurons are genotype specific and absent in *MAPT*^V337M^ neurons.

## Graphical abstract

**Figure:**
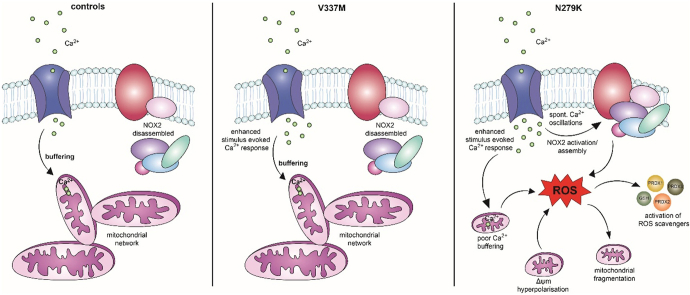

## Abbreviations

ΔΨm: mitochondrial membrane potential
CNS: central nervous system
FTLD: frontotemporal lobar degeneration
Glu/Gly: glutamate plus glycine
GO: gene ontology
GSH: genomic safe harbour
GSTP: glutathione S-transferase P
hiPSC: human induced pluripotent stem cells
MAPT: microtubule associated protein tau
MEA: multi electrode array
NOX: NADPH oxidase
PRDX1,2: peroxiredoxin 1,2
PRDX3: thioredoxin-dependent peroxide reductase
PSM: peptide spectra matches
ROS: reactive oxygen species
RT-qPCR: quantitative real-time polymerase chain reaction
smNPC: small molecule neural precursor cell

## 1 Introduction

Tauopathies are the most common type of proteinopathies underlying age-related neurodegenerative diseases [1]. Intracellular inclusions composed of abnormally modified microtubule-binding protein tau are found in a variety of subtypes of frontotemporal lobar degeneration (FTLD-tau), including Pick's disease, progressive supranuclear palsy, and corticobasal degeneration [2]. Moreover, neurofibrillary tau tangles are a defining feature of Alzheimer's disease neuropathology [3].

Tau is a neuronal microtubule-associated protein promoting microtubule assembly and cytoskeleton stabilisation. In humans, tau is encoded by the *microtubule associated protein tau (MAPT)*-gene [4]. In the adult CNS, six major isoforms are expressed by alternative splicing of exon (E)2, E3 and E10. Irrespective of the number of included N-terminal repeats, isoforms may contain either three or four C-terminal repeat domains (3R or 4R, respectively). The second of the four R repeats is encoded by E10 and is not integral part of 3R tau. The physiological expression of the six isoforms in the human CNS is developmentally regulated. In the early embryonic CNS only 0N3R, the shortest of all tau isoforms, is expressed. In contrast, in the adult CNS roughly equal amounts of 3R and 4R isoforms are expressed [[5], [6], [7], [8], [9]].

Today, >50 confirmed pathogenic mutations have been identified in the *MAPT* gene [10], causing hereditary FTLD-tau [11,12], which are subject to a high clinical variability spanning the entire clinical and pathological phenotypes associated with FTLD-tau [13]. Several mechanisms have been identified by which *MAPT* mutations may exert their pathogenic effects. They may either perturb RNA splicing, impair tau functions or promote tau fibrillation [14]. The N279K (exon 10) mutation impairs splicing of exon 10, resulting in a shift of the physiological 1:1 ratio of 3R and 4R tau isoforms towards a preponderance of the more aggregation prone 4R isoform [15]. Others, such as V337M (exon 12) cause a decrease in the *in vitro* affinity of tau to microtubules and a reduced ability to promote microtubule assembly [12,16,17]. V337M also renders tau a better substrate for tau protein kinases facilitating tau phosphorylation [18].

Most studies on the effect of mutant tau on neurons have focused mainly on mouse or rat tissue [19,20], reflecting the difficulty to obtain primary human brain tissue or robust and scalable quantities of human stem cell derived neurons. However, human model systems are important when studying the effects of *MAPT* mutations underlying hereditary FTLD-tau, since rodent neurons differ from their human counterparts regarding their tau composition [21,22]. This difference mainly affects the N-terminal end of tau, a binding site for proteins. Greatest binding to this site is seen for proteins involved in bioenergetics [23], which is an important process when studying neurodegeneration *in vitro*.

A previous study has shown that 4R tau exogenously applied to rodent neuronal cultures or human induced pluripotent stem cell (hiPSC) derived neurons carrying the intronic 10 + 16 *MAPT* mutation, known to lead to a tau-ratio shift towards the 4R isoform, is responsible for mitochondrial deregulation. In these neurons, calcium and hyperexcitability induced cell death was detected and thus 4R tau may contribute to neurodegeneration [20]. Using the same mutant hiPSC derived neurons, it was shown that mitochondria and reactive oxygen species (ROS) signalling play important roles in neurodegeneration *in vitro* [24].

Whether *MAPT* mutations that do not cause an increase of the 4R/3R ratio, affect the same signalling pathways remains to be determined. This is important since it may implicate that mutation specific pathways need to be addressed in rescue strategies and may imply a need for mutation specific therapies. It also remains to be determined whether findings in neurons with the intronic 10 + 16 *MAPT* mutation can be replicated in other mutations shifting the physiological 4R/3R ratio and whether these also prove robust when using isogenic controls.

Here, we present an optimised forward programming protocol for the robust and highly efficient production of pure cultures of glutamatergic cortical neurons from hiPSCs. We apply this protocol as a reductionist human *in vitro* model of FTLD to characterise the molecular and cellular effects of two distinct *MAPT* mutations causing hereditary FTLD-tau. Using an unbiased proteomics approach, we show that cortical neurons express *MAPT*-genotype-dependent dominant proteome clusters regulating apoptosis, ROS homeostasis and mitochondrial function. Moreover, live cell imaging demonstrates that both *MAPT* mutations affect excitability of membranes as reflected in spontaneous and stimulus evoked calcium signals albeit with more prominent changes seen in neurons with the N279K mutation (*MAPT*^N279K^ neurons). These calcium transients in *MAPT*^N279K^ neurons lead to hyperpolarisation of mitochondria and mitochondrial ROS production as well as ROS production through NADPH oxidase, changes that were not observed in neurons harbouring the V337M mutation (*MAPT*^V337M^ neurons) or in the isogenic control. Ultimately, these changes lead to cell death *in vitro* in *MAPT*^N279K^ neurons and upregulation of antioxidant proteins, supporting the pathological role of the increased 4R tau isoform for neuronal cell death and suggesting a *MAPT*-genotype specific effect on *in vitro* neurodegeneration.

## 2 Material and methods

### 2.1 Cloning sites and cloning strategy including templates

**Fig. 1:**
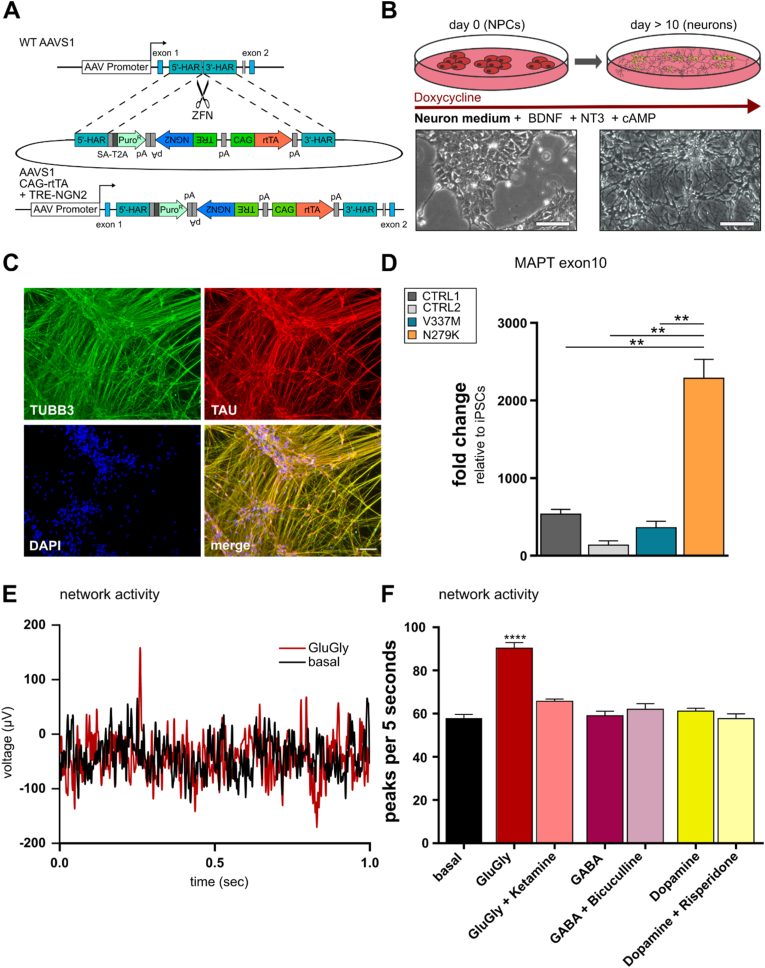
SmNPC targeting and induction to neurons. **A.** Schematic of AAVS1 targeting in smNPCs using an all-in-one AAVS1 donor vector containing a puromycin resistance cassette for antibiotic selection, both elements of the Tet-ON system in inverse directions and the inducible reprogramming factor *NGN2*. **B.** Schematic of the forward programming protocol for generating cortical neurons from smNPCs. Phase contrast images of smNPCs (day 0) and cortical neurons (day 14 after induction). **C.** ICC of cortical neurons after 10 days of induction using βIII-tubulin (*TUBB3*, green), TAU (*MAPT*, red), and DAPI for nuclei staining (blue). Scale bars: 100 μm. **D.** RT-qPCR showing the expression of exon10 of the *MAPT* gene. Fold changes are relative to hiPSCs. **E.** Representative measurements of MEA activity as measured in cortical neurons under baseline (black trace) and after pharmacological activation with glutamate/glycine (Glu/Gly, red trace). **F.** Bar graphs showing mean frequency of the field potential oscillations after different pharmacological treatment (shown on the x-axis). *p < 0.05; ****p < 0.0001. (For interpretation of the references to colour in this figure legend, the reader is referred to the Web version of this article.)

Based on our previously published AAVS1 donor vectors [25], we generated a novel AAVS1 donor plasmid containing both parts of the Tet-ON system in inverse orientation using Gibson Assembly. We inserted the coding sequence of *NGN2* as inducible transgene (Fig. 1A).

### 2.2 NPC culture and gene targeting

HiPSC-derived small molecule neural precursor cells (smNPCs) were obtained and cultured according to Ref. [26]. SmNPC lines were published previously and contributed by Tanja Kuhlmann [27,28]. In brief, smNPCs are maintained on Matrigel (354248, Corning) coated wells in smNPC media (1:1 DMEM/F12, Neurobasal Media, 1% l-Glutamine, 1% N2-Supplement, 1% B27-Supplement, 1% Penicillin/Streptomycin, 200 μM ascorbic acid, 0.5 μM SAG, 3 μM CHIR 99021) with media changes every 2–3 days. For gene targeting, we used an Amaxa Nucleofector II (Lonza) and the stem cell nucleofection kit (Lonza) following instructions. 2x10^6^ cells were transfected with 4 μg DNA per plasmid using program B-16. The novel all-in-one AAVS1 donor plasmid containing the inducible *NGN2* transgene was introduced into the AAVS1 locus using two zinc-finger nucleases (pZFN_AAVS1-L_ELD, pZFN_AAVS1-R_KKR). Antibiotic selection was performed using puromycin (A1113803, ThermoFisher, 0.75 μg/ml).

### 2.3 Forward programming of smNPCs into cortical neurons

Neuronal differentiation is induced by applying neuron media (DMEM/F12, 2% B27-Supplement, 1% N2-Supplement, 1% Penicillin/Streptomycin, 10 ng/ml BDNF, 10 ng/ml NT-3, 200 μM cAMP, 200 μM ascorbic acid, 1 μg/ml Doxycycline) and switching to half media change at day 10 after induction. Experiments were conducted between day 28 and 30 after induction, if not stated otherwise.

### 2.4 Immunocytochemistry

To evaluate the neuronal state after differentiation, cells were fixed using 4% paraformaldehyde (15 min, RT), washed 3x using DPBS [-]CaCl_2_ [-]MgCl_2_ (PBS−/−), blocked and permeabilised with a blocking solution (PBS−/−, 10% serum (Sigma Aldrich), 0.3% Triton X-100 (Sigma Aldrich) for 30 min, RT). Staining was performed overnight at 4 °C with either βIII-tubulin (TUBB3, 801201, Biolegend, 1:1000) or TAU (*MAPT*, A0024, Dako, 1:100). Secondary antibodies Alexa Fluor 488 (A-11001, Invitrogen^TM^, 1:500) and Alexa Fluor 594 (A-11012, Invitrogen^TM^, 1:500), respectively, were incubated for 1 h at room temperature. Finally, coverslips were mounted in fluorescent mounting medium containing 4’,6-diamidino-2-phenylindole (DAPI, 00-4959-52, Invitrogen^TM^). Visualisation was performed with a Keyence BZ-9000 BioRevo inverted microscope. Images were taken with a 20x objective, and a 100% digital zoom was applied to accentuate neuronal network structure.

### 2.5 Quantitative real-time PCR

Cells were collected in RLT buffer and total RNA was extracted with RNeasy Micro Kit (Qiagen). 500 ng of the isolated RNA was transcribed to cDNA with the Maxima reverse transcription series by ThermoFisher and 20 ng was used per reaction performing RT-qPCR with StepOnePlus Real-Time PCR System (Applied Biosystems). Analysis was conducted with StepOne software v2.1. The following primer pair was designed with the NCBI primer tool and measured with the fluorescent dye SYBR green: *MAPT*_exon10 (F-TCCAAGATCGGCTCCACTGA, R-CACACTTGGACTGGACGTTG). Expression levels of GAPDH (F-GTGAAGGTCGGAGTCAACGG, R-TGAAGGGGTCATTGATGGCA) served as an internal control in each sample. Values are shown as 2^−ΔΔCt^.

### 2.6 Electrophysiological characterisation of smNPC-derived neurons by microelectrode array (MEA)

Electrophysiological characterisation on microelectrode arrays (USB-MEA256system, Multichannel Systems) were performed by using 9-well MEA chips as previously described [29]. The MEA chips were plasma-cleaned and coated with a 1:75 Matrigel (Corning) dilution in KO-DMEM (Gibco^TM^) and 5% KSR (Thermo Fisher) overnight and additionally incubated with FBS (Sigma-Aldrich) at 37 °C for 30 min. Cell clusters were detached by using TrypLE (Gibco^TM^) transferred to the electrode area of the MEA chips and attached for about 96 h. The measurements of the cells were performed at a temperature of 37 °C. Neuronal activities were first recorded under basal conditions without any activator or inhibitor to record a reference signal. To identify neurotransmitter responsive network activity, different pharmacological agonistic and antagonistic modulators were applied to each sample chamber of a MEA chip and electric field potential was recorded. The activators glutamate/glycine (100 μM each, Sigma-Aldrich), dopamine hydrochloride (10 μM, Sigma-Aldrich) and GABA (10 μM, Sigma-Aldrich) were applied to selectively detect neurotransmitter responsive neuronal networks. After recording the activator responsive signals, selective inhibitors for the respective pathways were applied to the same well. The inhibitors used were risperidone (10 nM, Sigma-Aldrich), bicuculline (1 μM, Sigma-Aldrich) and ketamine (10 μM, Sigma-Aldrich). For recording the datasets, Cardio2D software (Multi Channel Systems MCS GmbH, Reutlingen, Germany) was used. Data was analysed using the softwares Cardio2D+ (Multi Channel Systems MCS GmbH, Reutlingen, Germany) and Origin v9.0 (OriginLab Corporation, Northampton, MA, USA). MEA analysis was performed on n = 4 independent replicates.

### 2.7 Proteome analysis

Normalisation: Cells were lysed in 200 μl lysis buffer consisting of 200 mM HEPPS in 8 M urea, pH 8.5 supplemented with 1x Halt Protease and Phosphatase Inhibitor (Thermo Fisher). Protein lysates were sonicated for 20 s on 10% power. After centrifugation of the samples at 1.400 rpm for 30 min at 4 °C, the supernatants were transferred to new tubes. Subsequently, protein concentrations were determined using a commercial BCA kit (Thermo Fisher). Protein adjusted sample aliquots were subjected to sodium dodecyl sulfate–polyacrylamide gel electrophoresis (SDS-PAGE) to fine-adjust protein amounts for label free proteome analysis. After staining the gel with Coomassie Blue according to manufacturer's protocol the optical density of each sample lane was determined with a calibrated gel scanner in transmission mode and the relative protein amount was calculated.

Digestion and fractionation: Sample preparation for mass spectrometry was performed via in-solution digestion and strong cation exchange (SCX) fractionation. In brief, samples were four-fold diluted in 25 mM NH4HCO3, pH 8.0, and subsequently incubated with 5 mM dithiothreitol at room temperature for 1 h. Afterwards, reduced cysteine residues were carbamidomethylated via addition of 20 mM iodine acetamide at room temperature for 1 h. Proteins were digested by adding 2.5 μg trypsin (TrypsinGold, Promega, Madison, WI, USA) and incubated at room temperature overnight. Digestion was stopped by adding formic acid (FAc) to a final concentration of 0.5% and subsequently centrifuged at 15.000×*g* at 4 °C for 15 min. Resulting supernatant was subjected to a SCX column (SCX SpinTips, Protea Biosciences, Morgantown, USA) previously equilibrated with 60 μl acetonitrile (ACN) and washed with 0.1% trifluoric acid (TFA). After sample application SCX column was washed with 60 μl 0.1% TFA. Fractionation was achieved by stepwise elution with 60 μl of: 50 mM ammonium formiate, 20% ACN, 0.5% FAc; 75 mM ammonium formiate, 20% ACN, 0.5% FAc; 125 mM ammonium formiate, 20% ACN, 0.5% FAc; 200 mM ammonium formiate, 20% ACN, 0.5% FAc; 300 mM ammonium formiate, 20% ACN, 0.5% FAc and 5% ammonium hydroxide, 80% ACN. Eluted fractions were dried in a vacuum centrifuge.

Mass spectrometry: LC-MS/MS was performed on a hybrid dual pressure linear ion trap/orbitrap mass spectrometer (LTQ Orbitrap Velos Pro, Thermo Scientific, San Jose, CA, USA) equipped with an Ultimate 3000-nLC Ultra HPLC (Thermo Scientific, San Jose, CA, USA). Dried peptide fractions were dissolved in 10 μL 0.1% TFA and subjected to a 75 μm I.D., 25 cm PepMap C18-column, packed with 2 μm resin (Dionex, Germany). Separation was achieved by applying a gradient from 2% ACN to 35% ACN in 0.1% formic acid (FA) over a 130 min gradient at a flow rate of 300 nL/min. The LTQ Orbitrap Velos Pro MS exclusively used CID-fragmentation when acquiring MS/MS spectra, consisting of an orbitrap full MS scan followed by up to 20 LTQ MS/MS experiments (TOP20) on the most abundant ions detected in the full MS scan. The essential MS settings were as follows: full MS (FTMS; resolution 60,000; *m/z* range 400–2000); MS/MS (Linear Trap; minimum signal threshold 500; isolation width 2 Da; dynamic exclusion time setting 30 s; singly charged ions were excluded from selection). Normalised collision energy was set to 35%, and the activation time was set to 10 ms.

Data processing: Raw data processing and protein identification of the high resolution orbitrap datasets were performed with de novo sequencing algorithms of PEAKS Studio 8.0 (Bioinformatics Solutions Inc., Waterloo, Canada) using the SwissProt database. The false discovery rate was set to <1%.

Raw data, complete identification listings and supplementary information are available at the ProteomeXchange Consortium via the PRIDE partner repository [[30], [31], [32]] with identifier PXD025063.

Label free quantification and GO-analysis: Label free quantification (LFQ) was performed using the algorithm integrated in PEAKS Studio 8.0 (Bioinformatics Solutions Inc., Waterloo, Canada) followed by a hierarchical clustering of significantly regulated proteins. Critical settings were for peptides: significance <0.05, spectral quality >0.5, avg. area>1E5; and for proteins: significance<0.01 (ANOVA), fold change>1.5, at least 3 unique peptides per protein.

GO-analysis of selected clusters was performed by filtering the unique annotated Gene Ontology terms for all proteins of each cluster. Percentage of coverage was calculated by counting the occurrence of the cluster specific terms divided by the number of proteins within the cluster. Resulting lists include the highest 10% of the unique terms only.

### 2.8 Live cell imaging and staining procedures

Live cell imaging was conducted with an epifluorescence-inverted microscope equipped with a 40x oil-immersion fluorite objective. Imaging was performed between 28 and 35 days after cells were induced and dyes were diluted in artificial cerebrospinal fluid (120 mM NaCl, 2.5 mM KCl, 1.25 mM NaH_2_PO_4_, 22 mM NaHCO_3_, 25 mM glucose, 2 mM CaCl_2_, 2 mM MgSO_4_). Fluorescence data was analysed with MetaFluor Fluorescence Ratio Imaging Software (Molecular Devices, LLC, Canada/US), Origin, Version 2019 (OriginLab Corporation, Northampton, MA, USA) and ImageJ 1.46r (Wayne Rasband, National Institute of Health, USA).

Fura-2 AM (F1221, Invitrogen^TM^) was used to measure cytosolic calcium. Cells were incubated with 5 μM of the dye for 30 min, washed once and images were taken every second. Ratiometric measurements were taken with 340 nm and 380 nm excitation light and emission was collected at 510/80 nm.

Cells were stained with 40 nM tetramethylrhodamine methylester (TMRM, T668, Invitrogen^TM^) for 40 min to assess mitochondrial membrane potential. The dye was kept present during the experiment and at least four regions of interest per coverslip were imaged with excitation at 530 nm and emission was collected at 705/72 nm.

To determine intracellular ROS production, cells were stained with 16 μM dihydroethidium (DHE, D11347, Invitrogen^TM^) and were instantly imaged with a frame interval of 5 s. Excitation light was provided at 530 nm and emitted light was detected at >590 nm. The dye was kept in the solution during image acquisition. We calculated rates of ROS production defined as increase of fluorescence over time (dF/dt).

Propidium iodide, which only permeates dead cells, and Hoechst, which stains all nuclei, were used in a co-staining to evaluate cell death. After 30 min incubation with the dyes (5 μM each) at least 4 ROI per coverslip were sampled. Excitation light was provided at 530 nm and 380 nm and emission filters at 705/72 nm and 510/80 nm were used respectively.

Experiments were repeated on at least three independent differentiation batches using two to three replicates per batch.

### 2.9 Morphological analysis of mitochondria

Neurons were co-stained with 20 nM Mitotracker (M22426, Invitrogen™) and 2 μM calcein AM (C3100MP, Invitrogen™) for 30 min to evaluate mitochondrial morphology. Images were taken with a Confocal Laser Scanning Microscope (Leica SP8) equipped with a 63x oil-immersion objective. Excitation light was provided by a HeNe laser at 633 nm and an Argon laser at 488 nm. Emitted light was detected with a photomultiplier module >670 nm and >500 nm respectively. Images were analysed using ImageJ 1.53d (Wayne Rasband, National Institute of Health, USA) as described by Ref. [33]. Representative images of the analysis strategy are shown in Supplementary Fig. 1A.

### 2.10 Flow cytometry

Apoptosis assay was performed with the FAM FLICA caspase 3/7 kit (ICT093, BioRad), which was used according to the protocol. Cells were incubated with the FAM-FLICA solution for 1 h at 37 °C and subsequently washed three times with 1X apoptosis buffer for 10 min at 37 °C. After detaching cells and creating a single cell solution, cells were resuspended in the fixative reagent. FACS analysis was conducted in a three-laser flow cytometer (Gallios, Beckman Coulter). Data analysis was performed using FlowJo™ v10.7.2 (FlowJo, LLC). The gating strategy is demonstrated in Supplementary Fig. 1B.

### 2.11 Statistics

Statistical analysis for all data except for the proteome analysis was performed using GraphPad Prism v6.01 (GraphPad Software Inc., CA, USA). Statistical significance was tested for using one-way ANOVA and post-hoc Tukey's test. For proteome analysis, statistical analysis was performed by post-hoc pairwise T-tests using the Benjamini Hochberg method for FDR correction with a significance level set <0.05.

## 3 Results

### 3.1 Deterministic programming of smNPCs into cortical neurons

We have developed a modified deterministic forward programming protocol based on forced expression of the transcription factor NGN2 in hiPSC-derived smNPCs to rapidly generate pure populations of cortical neurons. We previously reported that hiPSCs can be converted into cortical neurons by transient overexpression of NGN2 based on a dual genomic safe harbour targeting strategy of the Tet-ON system [25]. However, hiPSC cultures are labour-intensive and expensive, and compromised by endogenous epigenetic silencing mechanisms hampering the use of quicker inducible transgene expression systems. HiPSC-derived NPCs represent an intermediate stem cell population with unlimited self-renewal capacity and the developmental potency to differentiate into all major neuroectodermal cells of the brain that enable more convenient and cheaper culture conditions compared to hiPSCs.

Therefore, to obtain a scalable resource of cortical neurons, we have generated an all-in-one Tet-ON system AAVS1-targeting vector for inducible overexpression of NGN2 in smNPCs. Using zinc finger nucleases, the vector was inserted into the AAVS1 locus in a one-step targeting approach (Fig. 1A). Engineered smNPCs were induced with doxycycline in neuronal differentiation medium and exhibited axonal outgrowth within 2–4 days post-induction and rapidly formed neuronal networks (Fig. 1B). All cells express the pan-neuronal markers βIII-tubulin (*TUBB3*) and tau (*MAPT*) (Fig. 1C).

Next, we applied this targeting system to a previously published hiPSC/smNPC FTLD-tau model consisting of stem cells derived from patients with hereditary FTLD-tau due to the *MAPT*^N279K^ and *MAPT*^V337M^ mutations, respectively, alongside an isogenic control line of the *MAPT*^N279K^ mutation and an age-matched wild-type control. All targeted smNPC lines were rapidly converted into pure neuronal cultures. Further, we performed RT-qPCR analysis to characterise the tau isoform composition in cortical neuronal cultures using primer pairs specific to 4R tau transcripts. We observed a strong upregulation of 4R tau isoforms in cortical *MAPT*^N279K^ neurons compared to isogenic controls and *MAPT*^V337M^ neurons. At the same time, microelectrode array (MEA) analysis showed spontaneous electrical activity for neurons after 4 weeks of differentiation. This activity was significantly increased after adding glutamate plus glycine and decreased after adding ketamine, a specific NMDA channel inhibitor, suggesting that neurons are glutamatergic. When dopamine, risperidone, GABA or bicuculline were added, no significant activity changes could be observed (Fig. 1E-F). Based on these findings, further analysis was performed at day 28 of the novel smNPC forward programming protocol.

### 3.2 Proteome analysis reveals significantly up regulated ROS-relevant proteins in *MAPT*^N279K^ neurons

**Fig. 2:**
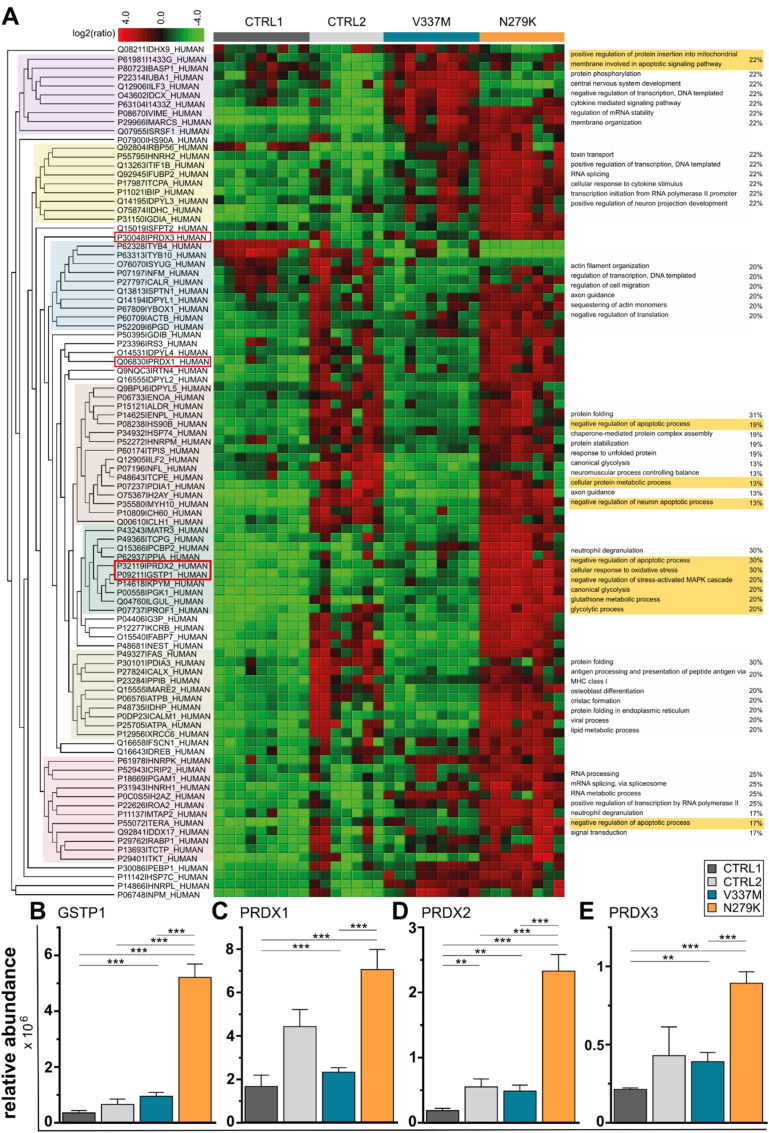
Mass spectrometry based proteomic comparative analysis of neurons. **A.** Cluster analysis of differentially expressed proteins among the neuronal cell lines: Label free quantification (LFQ) of high-resolution mass spectrometry data was performed on replicates of four different neuronal cell lines. Color-coded hierarchical clustering of significantly (ANOVA, p < 0.01) regulated proteins (left) and analysis by putative sharing of mutual GO terms in “biological processes” (right side of the figure). The percentage values refer to how many proteins of the cluster share the respective term. Clusters highlighted in yellow are GO terms concerned with apoptosis, ROS signalling and mitochondrial/metabolic function. **B-E.** Comparison of relative abundance of selected ROS-relevant proteins after proteome analysis: After large-scale proteome analysis of the four neuronal cell lines, ROS-relevant proteins within the differentially expressed group (ANOVA, p < 0.01) were selected for detailed comparison. This includes **B.** Glutathione S-transferase P (GSTP1), **C.** Peroxiredoxin-1 (PRDX1); **D.** Peroxiredoxin-2 (PRDX2) and **E.** Thioredoxin-dependent peroxide reductase (PRDX3). Statistical analysis was performed by post-hoc pairwise T-tests using the Benjamini Hochberg method for FDR correction. ***p < 0.001; **p < 0.005. (For interpretation of the references to colour in this figure legend, the reader is referred to the Web version of this article.)

For unbiased characterisation of *MAPT*-mutation-dependent cellular changes in cortical neurons, we performed proteome analysis using mass spectrometry (Fig. 2). Gene ontology (GO) terms that were prominent in *MAPT*^V337M^ neurons were involved in protein phosphorylation and cytokine mediated signalling pathways. First, we used the proteome data to characterise the differential regulation of tau phosphorylation. Proteome analysis of protein phosphorylation has shown that both mutants exhibit increased tau phosphorylation compared to controls. For instance, at Thr175 phosphorylation was only registered in mutant neurons, but not in controls. Moreover, while all genotypes were phosphorylated at Thr231, *MAPT*^N279K^ neurons were characterised by strikingly high peptide spectra matches (PSM), implying high levels of phosphorylation at this site compared to *MAPT*^V337M^ neurons and controls. Unique phosphorylation sites were seen at multiple serine residues in both mutants. *MAPT*^N279K^ neurons were phosphorylated at Ser237 and *MAPT*^V337M^ neurons showed PSM at Ser396, Ser400 and Ser404 whereas none of the other genotypes did (data not shown). Proteome data were supported by immunofluorescent stainings with the phospho-tau antibody AT8, detecting paired helical filaments, demonstrating a different subcellular distribution among different mutations with a prominent cytoplasmic distribution in *MAPT*^N279K^ neurons and a nuclear distribution in *MAPT*^V337M^ neurons and controls (Suppl. Fig. 2). Taken together, stronger phosphorylation in *MAPT*^N279K^ neurons together with prominent cytoplasmic phospho-tau localisation which differed from the nuclear phospho-tau localisation of *MAPT*^V337M^ neurons may imply that signaling cascades downstream of tau phosphorylation are differentially regulated in these two mutations.

In keeping with this hypothesis, we found that significantly regulated proteins clustered around GO terms associated with apoptosis, ROS homeostasis and mitochondrial function (Fig. 2A). *MAPT*^N279K^ neurons differed in the above-mentioned clusters when compared to *MAPT*^V337M^ neurons, suggesting that mutations may have different influences on apoptosis, ROS signalling and mitochondrial or metabolic function. Among the differentially expressed group of ROS-relevant proteins, glutathione S-transferase P (GSTP1), peroxiredoxin-1 and 2 (PRDX1, PRDX2) and thioredoxin-dependent peroxide reductase (PRDX3) were identified as significantly regulated and chosen for detailed comparison. GSTP1 protein abundance was almost 15-fold higher in *MAPT*^N279K^ neurons compared to controls (Fig. 2B). GSTP1 is involved in conjugation with reduced glutathione and many exogenous and endogenous hydrophobic electrophiles in antioxidant defence [34]. PRDX1, 2 and 3 catalyse the reduction of hydrogen peroxide to detoxify the cell from peroxides [35]. Their respective relative abundance was 4-, 12-, and 4-fold higher compared to controls (Fig. 2C-E). In keeping with the protein clustering heterogeneity of the two mutants, *MAPT*^V337M^ neurons did not show an upregulation of the respective proteins. The upregulation of these proteins that play a role in cell protection against oxidative stress might be interpreted as a rescue strategy for high ROS levels in *MAPT*^N279K^ neurons.

### 3.3 Neurons carrying *MAPT* mutations are more sensitive to glutamatergic signalling

**Fig. 3:**
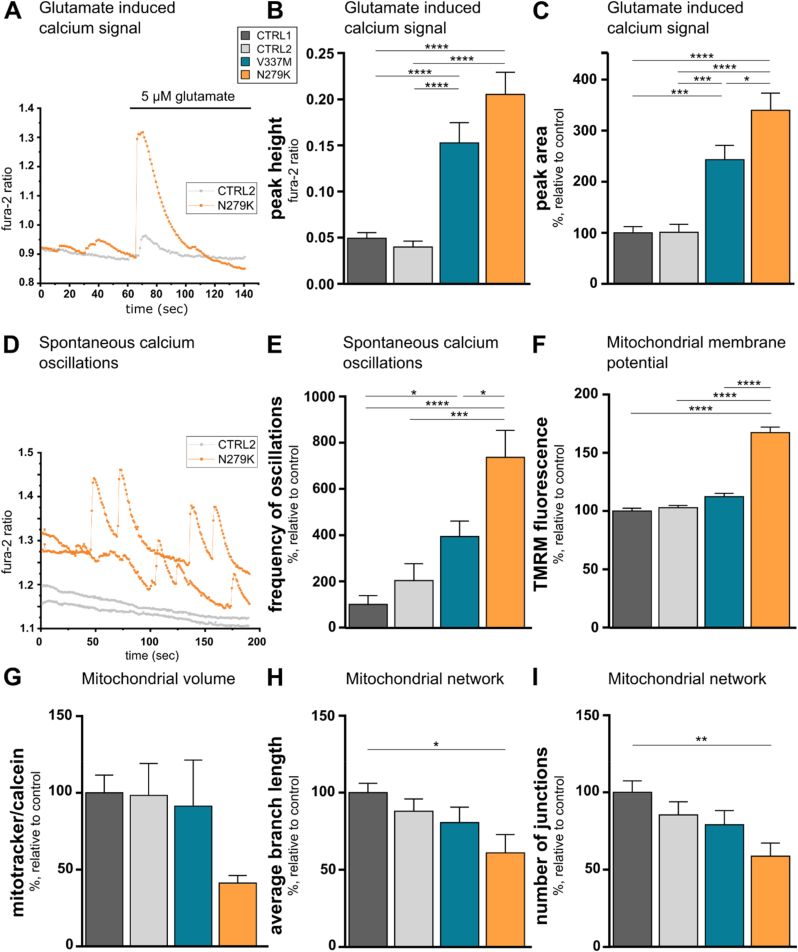
Aberrant glutamatergic signalling and mitochondrial changes in tau mutant neurons. **A.** Representative traces of control and *MAPT*^N279K^ neurons after physiological glutamate stimulation measured. **B.** Amplitude and **C.** peak area of glutamate evoked calcium signal in neurons as measured with Fura-2. **D.** Representative calcium trace under basal conditions of control and *MAPT*^N279K^ neurons. **E.** Frequency of spontaneous calcium oscillations measured with Fura-2 in neurons. **F.** Mitochondrial membrane potential evaluated with tetramethylrhodamine (TMRM) fluorescence intensity. **G.** Mitochondrial volume expressed as percentage of cell volume (evaluated with calcein staining) occupied by mitochondria (stained with mitotracker). **H–I.** Mitochondrial network analyses of neurons stained with mitotracker. *p < 0.05, **p < 0.01, ***p < 0.001, ****p < 0.0001.

A physiological hallmark of cortical neurons is their membrane excitability and information processing as well as excitotoxicity mediated through glutamate signalling. Particularly, the calcium response of neurons is acting in concert with increased ROS levels to facilitate ROS damage within the cell [36]. Under basal conditions, both types of mutant neurons showed significantly more spontaneous calcium oscillations compared to controls, which was even more pronounced in *MAPT*^N279K^ neurons versus *MAPT*^V337M^ neurons (Fig. 3D, E). Glutamate is the major excitatory neurotransmitter in the brain and low levels trigger calcium responses in neurons. The glutamate-induced calcium peak height and the area under the curve was significantly higher (*MAPT*^V337M^ 3-fold, 2.5-fold; *MAPT*^N279K^ 4-fold, 3-fold, respectively) in both mutants compared to controls (Fig. 3A-C). In addition, glutamatergic signalling via calcium abundance in the cytosol and subsequent buffering of calcium will also affect mitochondrial function.

### 3.4 Hyperpolarisation of the mitochondrial membrane potential, reduced mitochondrial mass, and increased mitochondrial fission in *MAPT*^N279K^ neurons

As neurons with *MAPT* mutations showed increased calcium responses (Fig. 3A-E), we also examined how this may influence the mitochondrial membrane potential (ΔΨm) as a universal indicator of mitochondrial health and function. Alterations of mitochondrial membrane potential in neurons are reported in several neurodegenerative diseases. In addition, mild calcium elevations are known to stimulate respiration, which in turn increases mitochondrial hyperpolarisation [37]. We found a hyperpolarised ΔΨm in *MAPT*^N279K^ neurons when compared to control and *MAPT*^V337M^ neurons suggesting altered mitochondrial function in these cells (Fig. 3F). Mitochondrial integrity affects morphology, content, and shape of mitochondria. In keeping with this, mitochondrial mass, expressed as the percentage of cell volume occupied by mitochondria normalised to control, of *MAPT*^N279K^ neurons was reduced by approximately 60% compared to controls and *MAPT*^V337M^ neurons (Fig. 3G). Mitochondria are dynamic cell organelles and undergo constant cycles of fusion and fission and an imbalance of these processes has been shown in AD and other neurodegenerative diseases with a shift towards more fragmented mitochondria in disease [38]. We found that the average branch length of mitochondria was reduced by 40% in *MAPT*^N279K^ neurons compared to controls (Fig. 3H). Concurrently, the number of junctions was over 40% higher in controls (Fig. 3I). These findings indicate a reduction of mitochondrial length and arborisation in *MAPT*^N279K^ neurons when compared to control cells.

### 3.5 N279K mutation leads to neuronal oxidative stress, which leads to cell death

**Fig. 4:**
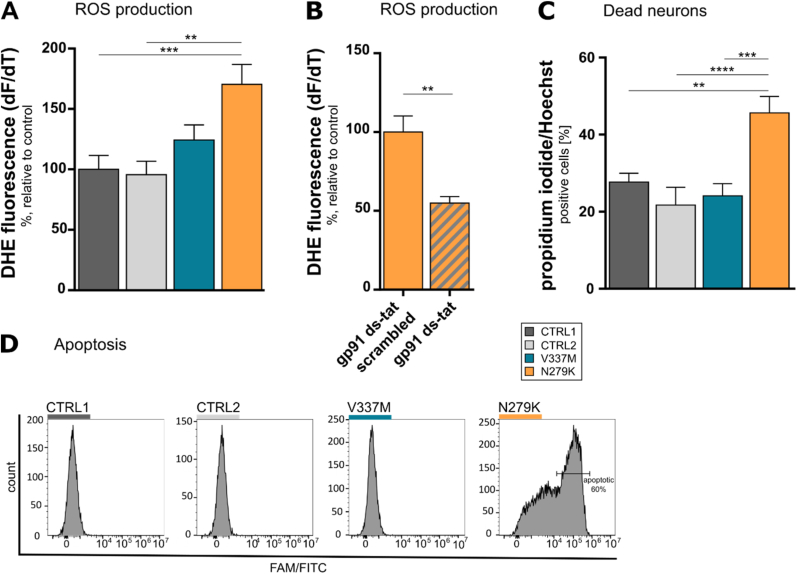
Impaired ROS homeostasis and increased cell death in *MAPT*^N279K^ neurons. **A.** Rate of ROS production measured with dihydroethidium (DHE). **B.***MAPT*^N279K^ neurons were pre-incubated with either gp-91 ds-tat or a scrambled form of the NOX2 inhibitor (50 μM, 30 min) with subsequent DHE measurement. Treatment groups were normalised to baseline DHE measurements of control (see **A**). **C.** Cell death analysis via co-staining of propidium iodide (stains only dead cells) and Hoechst (stains all cells). **D.** Flow cytometry analysis of apoptosis using caspase 3/7 staining. *p < 0.05, **p < 0.01, ***p < 0.001, ****p < 0.0001.

The initial unbiased proteomics indicated that proteins involved in ROS homeostasis were altered in *MAPT*^N279K^ neurons (Fig. 2). Thus, we speculated that ROS production within these cells might differ. Hyperpolarised mitochondria also lead to an increase in mitochondrial ROS production through an increase in the electron leak across the membrane. Therefore, we performed live cell imaging to examine the rate of neuronal ROS production and demonstrated that *MAPT*^N279K^ neurons had a 170% higher rate of ROS production compared to isogenic and wild-type controls, but also compared to *MAPT*^V337M^ neurons (Fig. 4A). A previous study suggested that NADPH oxidase, a key producer of ROS in neurons, might play a role in elevated ROS production due to insoluble tau aggregates [19]. We therefore incubated *MAPT*^N279K^ neurons with the NOX2 inhibitor gp91 ds-tat, which prohibits NADPH oxidase 2 assembly. Gp91 ds-tat reduced the rate of ROS production, thus identifying NADPH oxidase 2 as a substantial source of ROS in *MAPT*^N279K^ neurons (Fig. 4B).

To assess whether the above-mentioned aberrations in mitochondria, calcium signalling (Fig. 3) and ROS homeostasis (Fig. 4A, B) have incisive consequences for cell survival, we evaluated neuronal cell death. Co-stainings of propidium iodide and Hoechst revealed that *MAPT*^N279K^ neurons showed about 20% more cell death compared to controls and *MAPT*^V337M^ neurons (Fig. 4C). As we found alterations in mitochondria (Fig. 3F-I), which are a storage of caspases that trigger apoptosis, we also measured expression of caspase 3/7, which is involved in apoptosis. Flow cytometry analysis showed that 60% of *MAPT*^N279K^ neurons were positive for caspase 3/7 whereas the control lines and *MAPT*^V337M^ neurons showed no signs of apoptosis (Fig. 4D).

## 4 Discussion

In summary, we present a novel gene targeting strategy in human NPCs and an optimised cellular programming approach for robust and scalable production of pure bulk quantities of human cortical glutamatergic neurons. We applied this human cortical neuron model to analyse mutation specific effects of different *MAPT* mutations causing hereditary FTLD on mitochondria, ROS production and cell death. Cortical neurons harbouring the *MAPT*^N279K^ mutation, which causes aberrant splicing and increased production of the more aggregation prone 4R tau isoforms, were characterised by increased spontaneous and stimulus evoked calcium oscillations, mitochondrial dysfunction and increased ROS production ultimately leading to cell death. In contrast, cortical neurons carrying the *MAPT*^V337M^ mutation did not exhibit these changes.

Several previous studies have characterised neural stem cells and neurons derived from hiPSCs from patients with hereditary FTLD-tau caused by different *MAPT* mutations [9,24,27,[39], [40], [41], [42], [43], [44]]. All studies used classical differentiation protocols (e.g., Ref. [45] for the derivation of heterogenous populations of neural cell types with differing levels of neuronal purity, differing degrees of neuronal maturity, and different neuronal subtypes. Unsurprisingly, some basic neuronal phenotypes differed across studies and extremely long culture periods were required to reach the desired levels of neuronal maturity for cellular phenotyping [9,24].

From a technical perspective, classical neuronal differentiation protocols are lengthy and yield heterogeneous cell populations, lacking the maturity and purity required for robust downstream analysis. Two independent strategies have been thought of over the past ten years to overcome these problems. The first strategy was the capture of scalable intermediate populations during *in vitro* differentiation. To this aim, robust protocols for the derivation and maintenance of multipotent neural precursor cells (smNPCs) from hiPSCs using solely small molecule combinations have been established [26]. The use of hiPSC-derived smNPCs instead of hiPSCs as starting cell type for neuronal differentiation has several advantages: the maintenance culture is cheaper, less resource intensive, and less variable.

The second strategy was cellular reprogramming based on forced expression of master reprogramming factors. When combined with specific developmental extracellular cues, reprogramming enables high levels of precision for the making of specific target cell types. One of the prototypical and most applied programming protocols in stem cell research is the generation of glutamatergic cortical neurons by overexpression of NGN2 in hiPSCs using lentiviral vectors for transgene delivery and subsequent selection steps to enrich for successfully converted cells [46]. Recently, we have optimised this approach by using a dual genomic safe harbor (GSH)-based targeting system for controllable transgene expression in hiPSCs [25,47], enabling deterministic generation of cortical neurons without the need for viral gene delivery and without additional cell enrichment steps [25]. An unresolved issue and important bottleneck of our forward programming approach towards high-throughput and large screen applications are some of the characteristics related to hiPSC maintenance cultures: they are pricy, subject to variability, and resource intensive. Additionally, epigenetic silencing mechanisms in hiPSCs prevented the use of single GSH targeting strategies for inducible transgene expression [25].

Here, we combined both strategies and used smNPCs as expandable starting cell population for inducible forward programming into cortical neurons. To this aim, we constructed and targeted an “all-in-one” inducible NGN2 overexpression cassette into the AAVS1 locus of smNPCs, which enabled rapid and deterministic programming of smNPCs into cortical glutamatergic neurons upon transient transgene activation using doxycycline. Maturation kinetics appeared to be very fast, as demonstrated by morphological analysis, immunocytochemistry, and MEA analysis. Importantly, tau-isoform specific RT-qPCR demonstrated an early and robust shift in mutation-specific tau isoform expression, indicating the achievement of maturation levels that required several months when using classical differentiation approaches [9].

Cumulating evidence points towards a key role of cortical glutamatergic neurons in neurodegeneration in FTLD [48]. Most recently, an elegant study integrating transcriptomic and epigenomic analyses in post-mortem brain tissue, focusing on the main genetic subtypes of FTLD (*MAPT* and *GRN*), found that in these genetic subtypes excitatory neurons are the most affected cell type [49]. Similar to our findings, analyses in brain tissue of patients with hereditary FTLD-tau highlighted prominent roles and downregulation of pathways involved in energy metabolism, oxidative phosphorylation and organelle maintenance such as mitochondrial fusion and fission [38], however, *MAPT* mutation specific analysis has not been reported. Our finding that cellular dominant proteome clusters did not share many similarities, particularly with regards to clusters regulating apoptosis, ROS homeostasis and mitochondrial function, raises the awareness that different mutations within the same gene may have different effects and cellular downstream signalling cascades. This is in line with the clinical heterogeneity observed in patients with hereditary FTLD-tau, but the proposed high degree of clinico-genetic correlation depending on the type of *MAPT* mutation (Forrest et al., 2019).

We substantiated this hypothesis further using live cell imaging. Our findings of higher ROS production in *MAPT*^N279K^ neurons are further corroborated by results of a previous study that showed increased ROS production in neuronal cells derived from hiPSC from patients with hereditary FTLD carrying the intronic IVS10 + 16 *MAPT* mutation. The in depth study showed that mitochondrial ROS influence trafficking of the AMPAR and NMDAR subunits GluA1 and NR2B and increase their surface expression in neurons harbouring this mutation [58]. Similar to the N279K mutation, the *MAPT* IVS10 + 16 mutation leads to a tau isoform shift towards more 4R tau expression [24]. Importantly, the same group found that exogenous application of 4R tau reproduced the cellular changes observed in the IVS10 + 16 *MAPT*-mutant neurons [20] suggesting that 4R tau fragments trigger ROS production and metabolic changes. This would explain the absence of changes in ROS production in *MAPT*^V337M^ neurons as seen in our study and thus mutation-specific differences in the neuronal phenotype.

Given that ROS production has been linked to both neuronal firing [50] and mitochondria, and given the specific vulnerability of excitatory neurons in FTLD pathology, we speculated that neuronal activity is altered in *MAPT*-mutant neurons when compared to controls. We used calcium measurements as a surrogate of neuronal excitability to also establish a direct link to mitochondria, since mitochondria are considered the hub of calcium signalling and are directly affected by intracellular calcium. We found an increase in spontaneous calcium oscillations in both mutant cell lines paralleled by increased calcium responses evoked by a physiological calcium stimulus when compared to isogenic and non-isogenic controls. This increase in spontaneous calcium oscillations was more prominent in *MAPT*^N279K^ neurons when compared to *MAPT*^V337M^ neurons. These results suggest a higher level of excitability, which translates in more calcium influx into the cell. Mild intracellular calcium influx stimulates respiration. This in turn hyperpolarises the mitochondrial membrane potential, which is known to increase the generation of ROS [37]. This finding of increased excitability in neurons with *MAPT* mutations was corroborated by a previous study that provided insights into stimulus induced calcium responses in two different *MAPT* mutations (*MAPT* IVS10 + 14; *MAPT*^R406W^; [41]. In addition to stimulus evoked calcium oscillations, our study provides evidence for the occurrence of spontaneous, endogenous calcium oscillations.

We found lower mitochondrial mass together with hyperpolarisation of the mitochondria in *MAPT*^N279K^ neurons, which was not seen in *MAPT*^V337M^ neurons. Hyperpolarisation of mitochondria along with low mitochondrial volume has previously been described in neurons with *MAPT* mutations that cause a disbalance of the 3R–4R tau isoform ratio [24]. In this study, mitochondrial hyperpolarisation was considered the main source of cellular ROS production and would also explain ROS production in our *MAPT*^N279K^ neurons, which was paralleled by mitochondrial fragmentation in *MAPT*^N279K^ neurons, but not in *MAPT*^V337M^ neurons. Mitochondrial fragmentation is likely a result of increased ROS production in the cell. In addition to mitochondrial ROS, we were able to show that ROS production via NOX2 assembly plays a role in *MAPT*^N279K^ neurons. Previous studies have highlighted that glutamate can trigger ROS production and assembly of NOX2 [51]. We here show that spontaneous calcium transients lead to NOX2 assembly in *MAPT*^N279K^ neurons highlighting NOX2 as a potential treatment target.

Mitochondrial dysfunction and ROS production in *MAPT*^N279K^ neurons led to increased apoptosis and cell death in our study, which was not seen in *MAPT*^V337M^ neurons. Previous work using the same cell lines demonstrated that respiration inhibition with rotenone led to enhanced lactate dehydrogenase release in mutant neurons compared to controls, indicating that these cell lines are more vulnerable to exogenously applied stressors, ultimately leading to cell death [27]. However, pharmacological tools such as rotenone application are biased towards extreme toxicity - a stressor, which may not reflect the situation *in vivo* in FTLD. Neurodegeneration is a condition in which neurotransmitters and endogenous compounds play a key role, but it is not characterised by extreme pharmacological toxicity or excitotoxicity with very high levels of glutamate as it may occur in brain ischemia and modelled by excessive glutamate concentrations [52].

In our study, *MAPT*^V337M^ neurons did not show *in vitro* cell death when compared to control neurons. The discrepancy between a lack of a neurodegenerative phenotype *in vitro* and neurodegeneration, as seen in patients with this mutation, may relate to the absence of other cells such as microglia in our reductionist *in vitro* system, which have been highlighted as important players of neurodegeneration in FTLD [53,54]. Alternatively, it may implicate that additional exogenous stressors are required to induce neurodegeneration *in vitro* such as has been implied by previous studies [27,41].

Hereditary FTLD-tau represents a genotype-dependent pathologically and clinically heterogenous group of tauopathies. One of the potential causes of the observed heterogeneity is the brain region and/or neuronal subtype-dependent cellular vulnerability to mutation-specific mechanisms underlying the neurodegeneration. In the present study, we focused on mechanisms in cortical glutamatergic excitatory neurons, one of the major neuronal subtypes affected in FTLD. A strength of our study is the generation of pure cultures of this specific neuronal phenotype. Programming protocols for the precise derivation of other cellular phenotypes will help to delineate cell-type specific vulnerability. For instance, members of kindreds with the *MAPT*^N279K^ mutation are commonly affected by parkinsonism, in addition to cognitive deficits and behavioural changes. This is likely due to an involvement of midbrain i.e. the substantia nigra and basal ganglia [44,55] Therefore, it would be interesting to derive pure cultures of dopaminergic neurons and GABAergic inhibitory neurons, respectively, from hiPSCs with different *MAPT*-mutations. This would allow identification of neuronal subtype-specific vulnerability of mutation-specific mechanisms causing, for instance, parkinsonism in patients with the *MAPT*^N279K^ mutation. Previous studies reported the forward programming of dopaminergic neurons from hiPSCs by overexpression of ASCL1, NURR1, EN1, FOXA2, LMX1A, and PITX3 [56] while GABAergic neurons were generated by forced expression of ASCL1 and DLX2 (Yang et al., 2017). Interestingly, a previous study in rodent brain has found brain region specifity in ROS production and maintenance of redox balance with maximal rates of glutamate induced ROS production seen in midbrain [57]. This study highlights the differential effect of glutamate induced redox dysbalance of brain regions and thus also different cell types which are predominant in these regions. A study of the effect of glutamate induced ROS dysbalance in different neuronal cell types in *MAPT* mutations is desirable, but beyond the scope of this study which focussed on glutamatergic neurons.

In conclusion, we present a novel programming approach for the manufacture of pure bulk quantities of human cortical glutamatergic neurons with unprecedented speed and efficiency. Applying this approach for a reductionist FTLD-tau human *in vitro* model corroborated previous lines of evidence that glutamate signalling is key in neurodegeneration in FTLD-tau and redox disbalance underlies neuronal cell death. In an unbiased approach we identified ROS signalling and mitochondrial or metabolic function as key processes in *MAPT*^N279K^ neurons. Mechanistically, spontaneous calcium oscillations in *MAPT*^N279K^ neurons triggered mitochondrial hyperpolarisation and fission leading to mitochondrial ROS production, but also ROS production through NOX2 acting together inducing cell death. Importantly, our data show that these mechanisms are *MAPT* mutation-specific, supporting recent data from clinicopathological correlation studies that FTLD-*MAPT* is characterised by high clinical heterogeneity, but also by *MAPT*-mutation specific clinicopathological-genetic correlations.

## Funding

This work was supported by the fund “Innovative Medical Research” of the University of Münster Medical School (to MP; Project PA111614) and the German Research Foundation (DFG; to MP; Project PA2369/2-1).

## Declaration of competing interest

LK: none; AS: A patent application for the optimised forward programming protocol has been filed and submitted. CBS: none. LG: none. TK: none. AE: none. PD: none. MN: none. GS: none. SGM: none. HRS: none. HW: none. SK: none. MP: A patent application for the optimised forward programming protocol has been filed and submitted.
