# Supplementary material for: MAPT genotype-dependent mitochondrial aberration and ROS production trigger dysfunction and death in cortical neurons of patients with hereditary FTLD
Source: Redox Biol. 2022 Dec 30;59:102597. doi: 10.1016/j.redox.2022.102597 (PMC9817175; doi:10.1016/j.redox.2022.102597)
Supplement: Multimedia component 1 [file mmc1.docx]

**
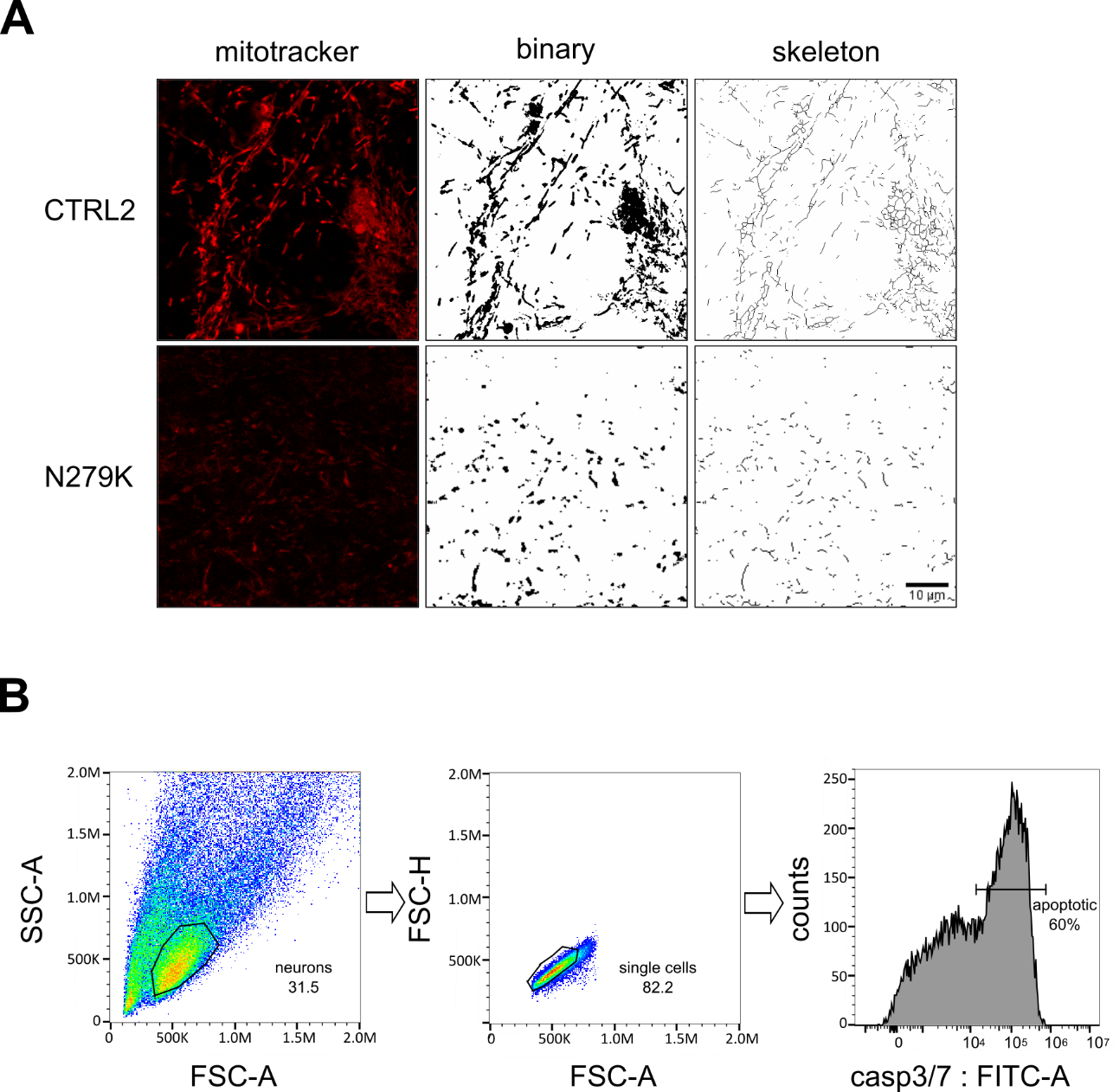
**

**Supplementary Figure 1. Mitochondrial network analysis and flow cytometry gating strategy.**


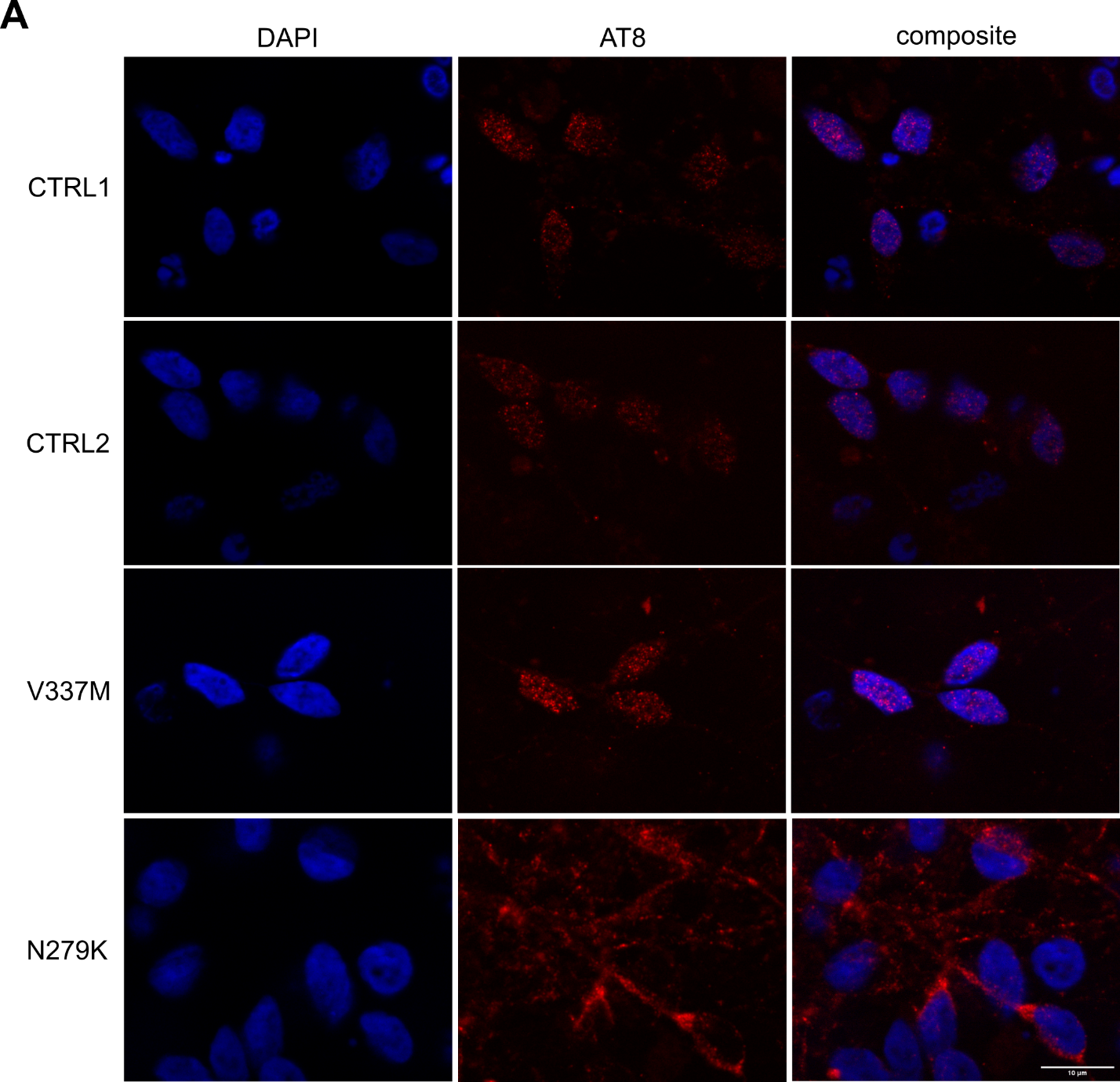


**Supplementary Figure 2. Hyperphosphorylated tau in patient-derived neurons.**
